# A Prototype for a Passive Resonant Interferometric Fiber Optic Gyroscope with a 3 × 3 Directional Coupler

**DOI:** 10.3390/s23031319

**Published:** 2023-01-24

**Authors:** Konstantin A. Ovchinnikov, Daniil G. Gilev, Victor V. Krishtop, Anatoliy B. Volyntsev, Vitaliy A. Maximenko, Alexey A. Garkushin, Yurii V. Filatov, Alexander S. Kukaev, Alexander A. Sevryugin, Egor V. Shalymov, Anastasiya V. Venediktova, Vladimir Yu. Venediktov

**Affiliations:** 1 Faculty of Physics, Perm State University, 614068 Perm, Russia; 2 Perm Scientific-Industrial Instrument Making Company, 614068 Perm, Russia; 3 Department of General Physics, Perm National Research Polytechnic University, 614990 Perm, Russia; 4 Laser Measurement and Navigation Systems Department, Electrotechnical University “LETI”, 197022 St. Petersburg, Russia; 5 Faculty of Physics, St. Petersburg State University, 199034 St. Petersburg, Russia

**Keywords:** fiber optical ring resonator, fiber optic gyroscope, polarization, resonant fiber optic gyroscope, 3 × 3 directional coupler, multibeam interference

## Abstract

Reducing the dimensions of optical gyroscopes is a crucial task and resonant fiber optic gyroscopes are promising candidates for its solution. The paper presents a prototype of a miniature resonant interferometric gyroscope of a strategic accuracy class. Due to the use of passive optical elements in this gyroscope, it has a great potential for miniaturization, alongside a low production cost and ease of implementation, since it does not require many feedback loops. The presented prototype shows results on a zero instability of 20°/h and an angle random walk of 0.16°/√h. A theoretical model explaining the nature of the multipath interference of resonant spectra and establishing the relationship between the resonator parameters and the output parameters of the presented prototype is proposed. The results predicted are in agreement with the experimental data. The prototype gyroscope demonstrates a scale factor instability and a change in the average signal level, which is due to the presence of polarization non-reciprocity, occurring due to the induced birefringence in the single-mode fiber of the contour. This problem requires further investigation to be performed.

## 1. Introduction

An optical ring resonator can be used as a sensitive element of a gyroscope in inertial navigation systems. Currently, several types of optical gyroscopes are used in inertial navigation, viz., ring laser gyroscopes (RLGs) and fiber optic gyroscopes (FOGs). FOGs and RLGs belong to the navigation accuracy class (angle random walk (ARW) is less than 0.01°/√h) and are used in groundborne, maritime, and air types of equipment. The rapid development of unmanned vehicles, robotic devices, and gyro-stabilized platforms of small sizes, results in a growing demand for small-sized and relatively inexpensive gyroscopes, a small-bodied MEMS gyroscope being among the latter. However, it has disadvantages limiting its use, such as worse sensitivity compared to optical systems (ARW > 1°/√h) and high sensitivity to vibration and shock impacts [1].

The reduction in the size of the fiber coil, the FOG-sensing element, leads to the deterioration in measurement accuracy caused by a decrease in the length and perimeter of the contour. A decrease in the overall dimensions of the RLG entails an increase in the dead zone of the gyroscope. A resonant contour of optical gyroscopes is a new step in the development of optical gyroscopy, as is evidenced by recent research and the development of resonant gyroscopes [2,3,4,5,6,7,8,9,10,11,12]. 

A potential configuration for a simple assembly of a gyroscope is a signal detection method using multipath white light interference and the correlation of two resonant spectra [5]. In the proposed configuration, the authors use phase modulation, which helps to achieve record performance for the resonant gyroscope, such as a bias instability of 0.009°/h and an ARW of 0.0093°/√h [6]. We have proposed a scheme for a completely passive and fiber resonant gyroscope by applying a constant phase shift, using a 3 × 3 directional coupler instead of a phase modulator.

## 2. The Sagnac Effect in a Ring Optical Resonator

In an optical ring resonator, two light waves propagate in opposite directions. At rest, each light wave will resonate at the same frequency. As the resonator rotates, the optical path difference resulting from the Sagnac effect will lead to a shift Δ*f*_S_ between the resonant frequencies of two counterpropagating waves. Thus, measuring the difference between the resonant frequencies of two counterpropagating signals Δ*f*_S_ allows you to determine the angular velocity of rotation of an object around an axis passing through the center of the fiber optic ring resonator (FORR), which is perpendicular to the plane of propagation of optical radiation in the FORR.

Various types of cavities (integrated optical, fiber optical, and whispering gallery mode resonators), which can be used as a sensitive element of a miniature optical resonant gyroscope are found in international publications [2,4]. The problem with implementing a resonant gyroscope is that it is not possible to directly measure the displacement of the resonant frequency; therefore, it is necessary to use various methods for detecting the angular velocity. In addition, the Sagnac effect is rather weak and contributes less to the shift of the resonant frequencies of the ring resonator than, for example, temperature changes.

The idea of detecting the angular velocity by locking the laser frequency at the resonant value using phase modulation was proposed in [10,11]. Then, the idea was developed in many papers, including the use of various types of resonators. Let us analyze the principle of operation of this method for detecting angular velocity.

**Figure 1:**
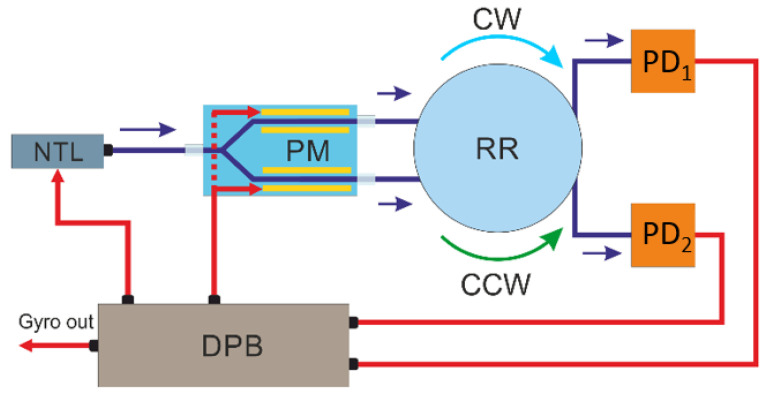
The scheme of the resonator gyroscope, implementing the principle of frequency synchronization. In the Figure: NTL—narrow tunable laser, PM—phase modulator, RR—ring resonator, PD—photodetector, DPB—digital processing board.

A narrow-band tunable laser with high-frequency stability is used to measure the frequency shift between two resonant frequencies clockwise (CW) and counterclockwise (CCW). The laser radiation frequency *f_L_* is kept at the resonant frequency *f_R_* of one of the directions (for example, CW) using a feedback loop. The value of the laser frequency adjustment is determined by the shift of the resonant peak of the *f_R_*. The scheme of a resonant feedback gyroscope is shown in Figure 1.

To track the offset of the resonant frequency, triangular phase modulation can be used, which creates a positive offset on one half-cycle (voltage increase) + *f*_m_, and on the other half-cycle (voltage decrease) − *f*_m_. Using an electro-optical modulator, two optical signals are formed with frequencies *f*_L_ ± *f*_m_, which are introduced into the optical resonator and form two oppositely directed electromagnetic waves (CW and CCW). After passing through the optical resonator, the radiation enters the photodetectors PD_1_ (for the CCW beam) and PD_2_ (for the CW beam). Signals from PD_1_ and PD_2_ are demodulated by a lock-in amplifier in the processing board, and the decoded signal from PD_1_ carries information about the amount of laser adjustment and thus forms a feedback loop. The demodulated signal from PD_2_ is translated into the value of the angular velocity.

The lock-in amplifier method can be used to demodulate the signal and determine the amplitude value of the square wave [12]. A rectangular signal *U*(*t*) with a certain amplitude Δ*U*_0_ and frequency ω, obtained from PD, is multiplied with a triangle signal *M*(*t*) from the generator. The result is passed through a low-pass filter to “subtract” the high-frequency component 2ω. As a result, the output remains a constant signal with a voltage equal to Δ*U*_0_, multiplied by the phase difference between the oscillator reference signal and the useful square wave. In the case of an optical resonant gyroscope, the signals are in-phase, so the lock-in amplifier generates a constant signal, the value of which corresponds to the amplitude of the received signal. Table 1 presents a selection of several works on the study of a resonant optical gyroscope with various types of resonators and their resonant and accuracy parameters.

To apply this scheme in practice, a highly stable tunable narrow-band radiation source is required, with the instability of the emitted frequency much smaller than the width of the resonant peaks of the studied resonators. In addition, it is necessary that the resonator be thermally stabilized for the feedback to work correctly. In practice, such conditions are difficult to fulfill because when adjusting the frequency of the laser, the resonance frequency goes away due to external influences at a much higher speed than the signal processing speed for tuning. The solution to the problem can be the use of large and expensive radiation sources and an increase in the overall dimensions of the optical gyroscope due to the need to use thermal insulation materials. In addition, a narrow-band laser is highly coherent, and as a result, parasitic effects appear in the optical scheme, complicating the measurement of angular velocity; for example, Rayleigh backscattering and the Kerr effect [2].

The solution to these problems is the use of a broadband radiation source; for this, an interference detection method with a 3 × 3 passive splitter is used in a resonant gyroscope.

## 3. Operating Principle

**Figure 2:**
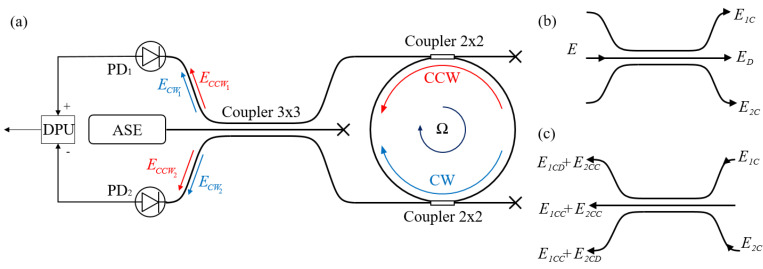
(**a**) The schematic diagram of the passive resonant fiber optic gyroscope with a 3 × 3 directional coupler. ASE—spontaneous emission amplifier; PD_1_ and PD_2_—photodetectors; DPU—signal-processing module, CW and CCW—irradiation passing through clockwise and counterclockwise, respectively (the rotation axis is normal to the picture plane; the rotation direction is clockwise); (**b**) the scheme of the radiation passage through the directional couple from ASE to the resonator; and (**c**) the scheme of the radiation passage through the directional couple from the resonator to the photodetectors.

Figure 2a shows the proposed scheme of the resonant interferometric fiber optic gyroscope (RIFOG). 

This contour consists of a spontaneous emission amplifier, the radiation of which passes through a 3 × 3 directional coupler and enters a FORR formed by a fiber coupler. The accumulation of the Sagnac phase occurs in the resonator for a certain set of frequencies. The radiation from the resonator again passes through the 3 × 3 coupler and subsequently interferes with the photodetectors PD_1_ and PD_2_. Let us describe the propagating radiation.

The irradiation from ASE complies with:

(1) $$E(\omega )=E_{0}(\omega )\exp\{i(\omega t+\varphi_{0})\},$$

where *ω* is cyclic frequency of radiation, *φ*_0_ is the initial phase, to simplify future calculations *φ*_0_ = 0, *E* is electric field intensity, and *E*_0_ amplitude of electric field intensity.

This field, passing through the directional coupler 3 × 3, propagates as is shown in Figure 2b, where:

(2) $$E_{D}=\frac{1}{\sqrt{3}}E_{0}\exp\left\{i\left(\omega t-\frac{\pi }{2}\right)\right\},$$

(3) $$E_{1C}=E_{2C}=\frac{1}{\sqrt{3}}E_{0}\exp\left\{i\left(\omega t+\frac{\pi }{6}\right)\right\},$$

the indices *D* and *C* denote the states for straight and crossed beams, respectively. Typically, for 3 × 3 couplers, the forward field collects a −π/2 phase delay, while the two crossed fields collect a π/6 delay with respect to the input field. Ideally, the power is divided equally among the three channels [16]. These formulae are considered valid for inputs on both sides of the coupler.

The fields *E*_1*C*_ and *E*_2*C*_ fall into the FORR and lay upon the fields propagating inside the resonator. Between the fields propagating in opposite directions inside the FORR, a Sagnac phase difference arises:

(4) $$\Delta \varphi_{s}=\frac{2\pi }{\lambda }\frac{LD}{c}\Omega ,$$

where Ω is angular velocity of rotation, *L* is the length of the FORR contour, *D* is the FORR diameter, *λ* is the central wavelength of the radiation, and *c* is the speed of light. 

Thus, the input fields of the resonator can be described as:

(5) $$E_{CW}=\frac{1-R}{\sqrt{3}}E_{0}\exp\left[i\left(\omega t+\frac{7\pi }{6}\right)\right]\sum_{q=0}^{\infty }R^{q}\exp\left\{i\left(\omega T+\frac{\Delta \varphi_{S}}{2}\right)q\right\},$$

(6) $$E_{CCW}=\frac{1-R}{\sqrt{3}}E_{0}\exp\left[i\left(\omega t+\frac{7\pi }{6}\right)\right]\sum_{p=0}^{\infty }R^{p}\exp\left\{i\left(\omega T-\frac{\Delta \varphi_{S}}{2}\right)p\right\},$$

where *R* is the output ratio by the power of the fiber couplers comprised in the FORR, *T* = *Ln/c_0_* is the FORR circuit bypass period, *n* is the refractive index of the fiber, *c_0_* is the speed of light in vacuum, *q*, and *p* is the number of contour bypasses for CW and CCW waves, respectively.

The fields, repeatedly passing through the 3 × 3 coupler, and propagating according to the scheme shown in Figure 2c, accumulate a phase delay, which depends on the propagation path inside the coupler; then, the fields are superposed. The result of this interference, taking into account (5) and (6), will be written as:

(7) $$E_{1}=E_{CW_{D}}+E_{CCW_{C}},$$

(8) $$E_{2}=E_{CW_{C}}+E_{CCW_{D}},$$

(9) $$E_{2}=E_{CW_{C}}+E_{CCW_{C}}.$$

The resulting field *E*_3_ does not participate in the interference to follow-up; therefore, it is not considered. 

The characteristics of the fiber resonator are mainly determined by two parameters: the length of the fiber contour *L* and the output ratio *R* of the two fiber couplers. If these characteristics are known, parameters *FSR, FWHM,* and *F* (Finesse) can be calculated using the following formulae:

(10) $$FSR=\frac{c}{nL}[\mathrm{Hz}],\;FWHM=\frac{(1-R)c}{\pi \sqrt{R}nL}\;=\;\frac{\left(1-R\right)}{\pi \sqrt{R}}FSR\;[\mathrm{Hz}],\;F=\frac{FSR}{FWHM}=\frac{\pi \sqrt{R}}{\left(1-R\right)}.$$

Taking into account that *T =* 1/*FSR* switches from a cyclic frequency of radiation *ω* to a frequency of *f* = *ω/*2*π*, in fields *E*_1_ and *E*_2_, one has:

(11) $$E_{1}(f)=-\frac{2(1-R)}{3}E_{0}(f)\sum_{q=0}^{\infty }R^{q}\exp\left\{i\frac{2\pi f}{FSR}q\right\}\cos\left(\frac{\Delta \varphi_{s}}{2}q+\frac{\pi }{3}\right),$$

(12) $$E_{2}(f)=-\frac{2(1-R)}{3}E_{0}(f)\sum_{p=0}^{\infty }R^{p}\exp\left\{i\frac{2\pi f}{FSR}p\right\}\cos\left(\frac{\Delta \varphi_{s}}{2}p-\frac{\pi }{3}\right).$$

The power of radiation incident on photodetectors 1 and 2 will be:

(13) $$P_{1}(f)=\int_{f_{1}}^{f_{2}}E_{1}(f)E_{1}^{*}(f)df=\frac{4{\left(1-R\right)}^{2}}{9}P_{0}\sum_{m=0}^{\infty }R^{2m}\cos^{2}\left(\frac{\Delta \varphi_{s}}{2}m+\frac{\pi }{3}\right),$$

(14) $$P_{2}(f)=\int_{f_{1}}^{f_{2}}E_{2}(f)E_{2}^{*}(f)df=\frac{4{\left(1-R\right)}^{2}}{9}P_{0}\sum_{m=0}^{\infty }R^{2m}\cos^{2}\left(\frac{\Delta \varphi_{s}}{2}m-\frac{\pi }{3}\right),$$

where ^*^ is the complex conjugate, *P*_0_ is output power ASE, and *m* is the number of contour bypasses when *p* = *q*. The power difference at the photodetectors will conform to:

(15) $$\Delta P=P_{1}-P_{2}=\frac{2\sqrt{3}{\left(1-R\right)}^{2}}{9}P_{0}\sum_{m=0}^{\infty }R^{2m}\sin\left(\Delta \varphi_{s}p\right).$$

here Δ*P* = ∑*R*^2*m*^sin(Δ*φ_s_m*) is the Fourier series in sines, which reduces to the function sin(Δ*φ_s_*)/(*R*^2^ − cos(Δ*φ_s_*) + 1/*R*^2^). Thus, (15) can be written as:

(16) $$\Delta P=\frac{2\sqrt{3}}{9}P_{0}\frac{{\left(1-R\right)}^{2}}{R^{2}-2\cos(\Delta \varphi_{s})+1/R^{2}}\sin(\Delta \varphi_{s}).$$

Similarly to (16), the Fourier series in (13) and (14) can be replaced by the following functions:

(17) $$P_{1}=\frac{{\left(1-R\right)}^{2}}{9}P_{0}\left[\frac{2}{1-R^{2}}+\frac{R^{2}(\cos\Delta \varphi_{s}-\sqrt{3}\sin\Delta \varphi_{s})-1}{R^{4}-2R^{2}\cos\Delta \varphi_{s}+1}\right],$$

(18) $$P_{2}=\frac{{\left(1-R\right)}^{2}}{9}P_{0}\left[\frac{2}{1-R^{2}}+\frac{R^{2}(\cos\Delta \varphi_{s}+\sqrt{3}\sin\Delta \varphi_{s})-1}{R^{4}-2R^{2}\cos\Delta \varphi_{s}+1}\right].$$

## 4. Numerical Simulation Results

**Figure 3:**
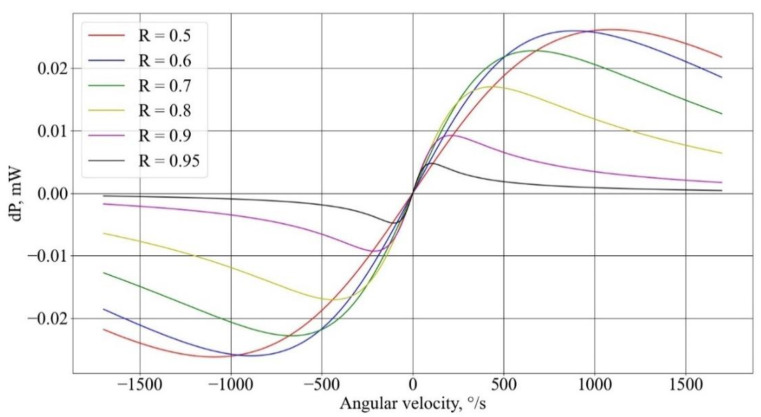
Dependency of the power difference upon the magnitude of the angular velocity at different output ratios of the fiber couplers, forming the resonator.

**Figure 4:**
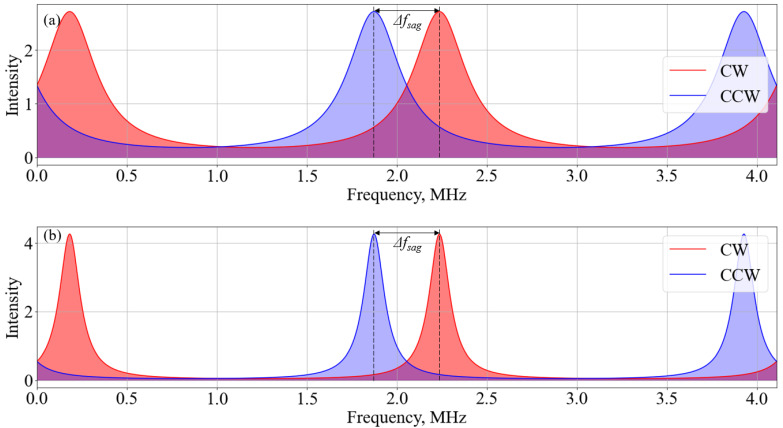
The frequency detuning resonance spectra when rotation takes place (**a**) at output ratio R = 0.6 and (**b**) at output ratio R = 0.9.

A numerical study of the influence of the resonator parameters on the characteristics of the gyroscope was carried out in the Python programming language using Formulas (16)–(18). The resulting dependencies for fiber length 50 m (Figure 3) of the signal power difference upon the Sagnac phase at different output ratios *R* of the fiber couplers forming the resonator show that with an increase in *R*, the range of the linear section decreases. This is due to the fact that with an increase in *R*, the *FWHM* value decreases with *FSR* being constant, which results in a diminishing of the interference region of the resonance spectra (Figure 4), with a frequency shift caused by rotation due to the Sagnac effect.

Figure 3 also shows that as *R* increases up to 0.6, the inclination angle of the linear section increases, and when *R* is greater than 0.6, the inclination angle practically does not change, owing to a decrease in the fraction of the useful signal reaching the photodetectors. Therefore, the coefficient *R* = 0.6 is the optimal one in terms of losses and the slope of the linear section.

## 5. Description of the Experiment and Measured Values of the Gyroscope Parameters

A further experiment demonstrates the multipath interference of a broadband source for a FORR of various lengths (65 m, 100 m, and 200 m). The FORR is made of two fiber couplers and a fiber coil, 50 mm in diameter. The entire scheme shown in Figure 1 is built on a single-mode fiber. Using the scanning method of a narrow-band laser, taking into account the nonlinearity of radiation frequency tuning [17,18], we measured the parameters of the FORR with fiber coils of various lengths. The data are shown in Table 2.

**Figure 5:**
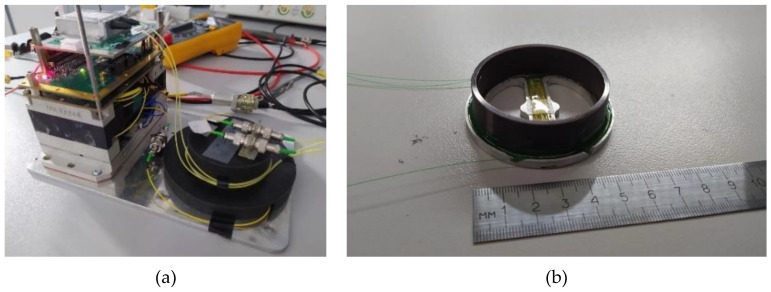
(**a**) Experimental setup, realizing the scheme of the resonant interferometric gyroscope with a 3 × 3 directional coupler and (**b**) a model of the sensing element of the optical resonant gyroscope with overall dimensions Ø60 × 25 mm.

To run an experiment aimed to determine the gyroscope parameters, a spontaneous emission amplifier manufactured by Perm scientific–industrial instrument-making company (Russia), and two photodiodes (PDI-20-P10-20G-W Laserscom, Belarus) with a transimpedance amplifier and a processing board with subtraction of two electrical signals were used. The ASE radiation power was 15 dBm, the photosensitivity was chosen to be the same and equal to 0.9 A/W. The optical scheme is assembled on single-mode fiber SMF-28Ultra Corning. Fiber dividers were produced by Perm scientific–industrial instrument-making company (Russia). A photo of a research stand with a sensing element is shown in Figure 5.

To measure the scale factor (*SF*), the developed stand is placed on a rotating platform TES-3V. The research stand periodically rotates at a speed of Ω = ±1°/s. The output signal value is registered in clockwise U_+_ and counterclockwise U_–_ rotation regimes. Then, the average value of SF for several measurements *N* is calculated according to the constraint:

(19) $$SF=\frac{\sum_{i=1}^{N}\left(\frac{\left|U_{i+}-U_{i-}\right|}{2}\right)}{N}.$$

**Figure 6:**
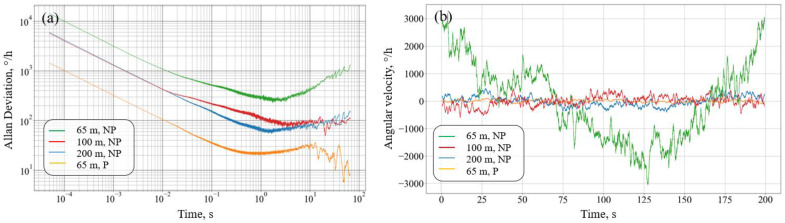
(**a**) Allan variation at different resonators and (**b**) the recording of RIFOG signals at different resonators; NP—polarization mismatching and P—matched polarization.

To measure zero instability and random angular drift, the signal is recorded for a long time at a frequency of 20,000 Sa/s with a duration of 200 s. Thus, a data array of 4 million points in length is formed for each FORR. Based on the resulting array, the Allan variation is constructed for each time window; Figure 6a shows the Allan deviation for various averaging times. Figure 6b represents the recording of the signal when averaged with a time window of 1 s.

The obtained dependencies show that the assembled circuit contains several types of noise, including random walk of the angle (white noise) and random walk of the angular velocity (Brownian noise). It is also possible to observe attaining the horizontal plateau; however, this does not mean that the level of flicker noise is reached since in terms of voltage, the level of pink noise is much lower (<1°/h). The FORR parameters obtained during the experiment are shown in Table 3. The table also lists theoretical estimates of the ARW value based on the equations from [22], taking into account the proposed theoretical model of a resonant gyroscope.

## 6. Noise Analysis

An ideal single-mode fiber has no birefringence and maintains any polarization state entered into the fiber without change. Real fibers have some anisotropy associated with the violation of the circular symmetry of the fiber, or with an asymmetric stress field in the cross-section of the fiber, which leads to the occurrence of birefringence.

**Figure 7:**
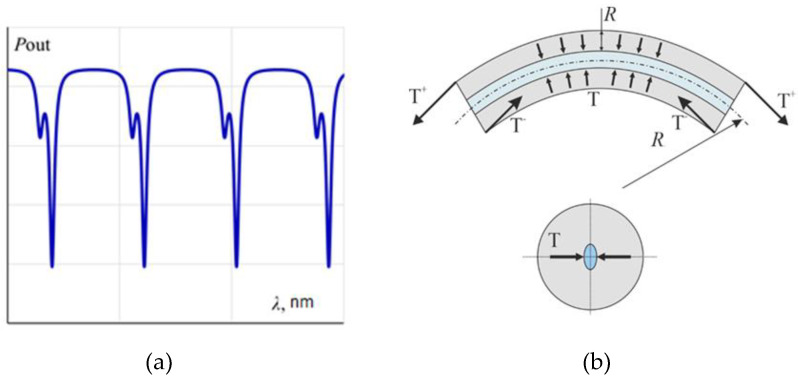
(**a**) Resonance spectrum for induced birefringence in the resonator contour and (**b**) core compression caused by bending deformation.

The main problem of the application of this scheme is appearing the polarization non-reciprocity because of is by the presence of birefringence in the contour of the resonator on a single-mode fiber. Birefringence in the resonator is induced by core deformation caused by fiber bending [19]. It results in a different propagation velocity of two polarization modes in the fiber and the appearance of an additional resonant peak (Figure 7) [20,21,22,23].

**Figure 8:**
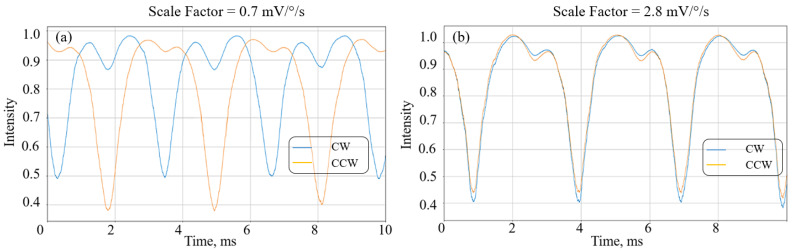
Resonant structures; induced birefringence is taken into account in (**a**) polarization mismatching and (**b**) matched polarization.

Insofar as during assembly the input fiber in the FORR can be deformed and twisted, radiation with one polarization state can enter the FORR in the clockwise direction, and the opposite direction with another polarization state. The absence of any interference caused by the non-correlation of the two resonant spectra can occur at the output. Due to the decrease in the value of the correlation of two resonances, the SF and the sensitivity of the gyroscope decrease, which can be seen for a 65 m long FORR with different input polarization states (Figure 8). 

For this case, separate records at different values of SF were completed; as a result, an increase in the general noise level and the presence of a trend in angular velocity can be observed on the Allan variation and the averaged long-term graph. This is due to both the appearance of birefringence in the FORR and the temperature impact, which leads to the redistribution of power between resonant frequencies and the deterioration of the accuracy of the gyroscope. 

The following ways to reduce the influence of polarization nonreciprocity are possible:

- Incorporation of optical polarizers before entering the FORR; however, this complicates the proposed design;
- Creation of components on an anisotropic fiber and the transition of the optical scheme to an anisotropic fiber;
- Embedding of optical depolarizers after the laser.

The configuration of the optical gyroscope with a 3 × 3 coupler and the FORR on a single-mode fiber would cater to the construction of simple gyroscopes of medium accuracy (<10°/h), but suits only short-term operation. In the case of a long-term operation regime, a big accumulation of errors in the angular velocity occurs. To increase the accuracy up to the level of 1°/h and the stability of gyroscope operating, it is necessary to reduce the effect of polarization non-reciprocity.

## 7. Conclusions

A simple configuration of the miniature fiber optic interferometric resonant gyroscope with a zero instability of 20°/h and an angle random walk of 0.16°/√h in overall dimensions of Ø60 × 25 mm and a fiber contour length of 65 mm is proposed. The proposed scheme can potentially be cheaper than a resonant gyroscope using a narrow-band laser, due to the use of simple passive elements and the absence of a complex signal processing system.

It has been established that the effect of induced birefringence in the single-mode fiber leads to the degradation in the scale factor when the input polarization states mismatch in an optical resonant gyroscope. Further study of this scheme should be directed to improving the accuracy up to the level of 1°/h and the stability of the gyroscope output signal. 

This research was carried out with the financial support of the Ministry of Science and Higher Education of the Russian Federation in the framework of the program of activities of the Perm Scientific and Educational Center “Rational Subsoil Use”.

## Figures and Tables

**Table 1:** A comparative table of accuracy parameters of sensitive elements from various works.

Resonator Type	Size of the Sensing Element	*F*	BI, °/hour	*ARW*, °/√hour
Whispering gallery mode resonator [13]	*D* = 0.007 m	200	3	0.02
Integral [14]	*S* = 2.10^−6^ m^2^	–	21,600	650
Fiber optic [15]	*D* = 0.05 m, *L* = 0.6 m	202	9.6	0.64

**Table 2:** Parameters of the FORR on a single-mode fiber.

No. FORR	*L*_res_, m	*FSR*, kHz	*FWHM*, kHz	*F*
1	65	3190 ± 63	640 ± 8	4.98
2	100	1955 ± 29	474 ± 19	4.12
3	200	1077 ± 13	378 ± 18	2.84

**Table 3:** Accuracy parameters for each FORR.

Measurement	SF, mV/°/s	ARW, °/√h	BI, °/h	ARW_theory_, °/√h
65 m, NP	0.7	1.67	250	0.0253
100 m, NP	2.6	0.67	80	0.0162
200 m, NP	10.5	0.67	60	0.0106
65 m, P	2.8	0.16	20	0.0253

## Data Availability

Not applicable.
